# High-Performance Detection of Exosomes Based on Synergistic Amplification of Amino-Functionalized Fe_3_O_4_ Nanoparticles and Two-Dimensional MXene Nanosheets

**DOI:** 10.3390/s23073508

**Published:** 2023-03-27

**Authors:** Linlin Zhuang, Qiannan You, Xue Su, Zhimin Chang, Mingfeng Ge, Qian Mei, Li Yang, Wenfei Dong, Li Li

**Affiliations:** 1 School of Biomedical Engineering (Suzhou), Division of Life Sciences and Medicine, University of Science and Technology of China, Hefei 230026, China; 2 Institute of Biomedical Engineering and Technology, Chinese Academy of Sciences, Suzhou 215163, China; 3 Chongqing Institute of Green and Intelligent Technology, Chinese Academy of Sciences, Chongqing 400714, China

**Keywords:** exosomes, Ti_3_C_2_ MXene, magnetic nanoparticles, electrochemical biosensor, synergistic amplification

## Abstract

Exosomes derived from cancer cells have been recognized as a promising biomarker for minimally invasive liquid biopsy. Herein, a novel sandwich-type biosensor was fabricated for highly sensitive detection of exosomes. Amino-functionalized Fe_3_O_4_ nanoparticles were synthesized as a sensing interface with a large surface area and rapid enrichment capacity, while two-dimensional MXene nanosheets were used as signal amplifiers with excellent electrical properties. Specifically, CD63 aptamer attached Fe_3_O_4_ nanoprobes capture the target exosomes. MXene nanosheets modified with epithelial cell adhesion molecule (EpCAM) aptamer were tethered on the electrode surface to enhance the quantification of exosomes captured with the detection of remaining protein sites. With such a design, the proposed biosensor showed a wide linear range from 10^2^ particles μL^−1^ to 10^7^ particles μL^−1^ for sensing 4T1 exosomes, with a low detection limit of 43 particles μL^−1^. In addition, this sensing platform can determine four different tumor cell types (4T1, Hela, HepG2, and A549) using surface proteins corresponding to aptamers 1 and 2 (CD63 and EpCAM) and showcases good specificity in serum samples. These preliminary results demonstrate the feasibility of establishing a sensitive, accurate, and inexpensive electrochemical sensor for detecting exosome concentrations and species. Moreover, they provide a significant reference for exosome applications in clinical settings, such as liquid biopsy and early cancer diagnosis.

## 1. Introduction

Exosomes, a type of extracellular vesicle widely found in body fluids such as blood, urine, saliva, and breast milk, are about 30–150 nm in diameter. Exosomes were first discovered by Johnstone in 1983 in the reticulocytes of sheep [1]. However, at first, exosomes were thought to be only a way for cells to excrete waste. Later, G. Raposo et al. in 1996 discovered that exosomes, similar to B-lymphocytes, stimulate anti-tumor responses [2]. Subsequently, exosomes have been successively found to play an important role in cellular communication, tumorigenesis, and the development of neurodegenerative diseases [3,4]. Multiple studies have reported that exosomes may participate in tumorigenesis and metastasis by changing the tumor microenvironment [5], inhibiting the autophagy pathway [6], and intercellular communication between tumor cells [7]. Many surface proteins are distributed on the surface of exosomes, including conventional transmembrane proteins (CD9, CD63, and CD81) [8] and heat shock proteins. Moreover, the types and contents of surface proteins vary greatly among exosomes of different origins. Therefore, exosomes are critical in the search for new tumor markers and tumor diagnosis [9]. This approach avoids invasive trauma as well as provides new ideas and methods for liquid biopsy [10]. Exosomes have been investigated as drug delivery carriers for targeted drug therapy since they are tiny, non-toxic, and a normal component of cellular products [11]. The aforementioned discoveries and applications have gradually made exosome research a hot topic for disease diagnosis and treatment.

Currently, the methods for detecting exosomes are mainly focused on traditional molecular biology assays, such as dynamic light diffraction, flow cytometry, and ELISA [12,13,14]. Furthermore, some new detection platforms have been constructed using novel methods such as microfluidic chips [15], surface plasmon resonance [16], and Raman spectroscopy [17]. However, these methods have the disadvantages of high cost, complex operation, and high sample volume requirements. In contrast, electrochemical sensors have been widely used for detecting various biomarkers as well as toxic and hazardous pollutants in food safety and other fields for their high sensitivity, low price, and simplicity [18,19,20]. In the field of exosome detection using electrochemical sensors, Huang et al. constructed an electrochemical sensor for detecting gastric cancer using a specific combination of aptamers and exosomes combined with a signal amplification strategy [21]. Zhang et al. prepared a highly sensitive and selective electrochemical biosensor using MXene as a nanocarrier and generating PB in situ on its surface, thus further amplifying the electrochemical signal [22]. Although electrochemical sensors for exosome detection are constantly being developed, obtaining a highly sensitive, stable, rapid, convenient, and low-cost detection method remains important.

As one of the most widely used magnetic nanomaterials, Fe_3_O_4_ nanoparticles, with their high surface-to-volume ratio, biocompatibility, stability, and excellent magnetic properties, have been widely researched [23,24,25,26]. Currently, magnetic nanoparticles are mainly used within the fields of magnetic separation, photothermal therapy, drug carriers, bioimaging, and pollutant adsorption and removal [27,28,29,30,31]. Magnetic separation, with its advantages of rapidness and recyclability, is also used in the field of electrochemical sensing. Mohammad et al. developed a screen-printed electro-sensor based on Fe_3_O_4_@CNC/Cu composite for identifying urine, water, and drug formulations as well as detecting venlafaxine in samples with a detection limit of up to 0.01 µM [32]. Jeong et al. combined magnetic separation of exosomes and electrochemical detection to create a novel portable device [33].

MXene, as a new type of two-dimensional material, has the advantages of a large specific surface area, good hydrophilicity, excellent electrical conductivity, and rich surface groups [34,35,36,37]. The surface modification of MXene obtained by different synthesis strategies has different termination groups, which can flexibly change the mechanical strength and electrochemical properties, providing a basis for obtaining MXene materials with modifiable properties [38]. Many studies worldwide have reported that MXene has not only become an ideal material for manufacturing supercapacitors [39] and batteries [40] but also has been applied in optoelectronics and microelectronics [41], wireless communication [42,43], water pollution control [44], and other fields. In the field of electrochemical energy storage, MXene can be used as an active material for supercapacitors and as an electrode material for alkaline metal ion batteries due to its excellent rate performance, high capacities, high energy densities, adjustable interlayer spacing, Na^+^ and K^+^ intercalation, and good conductivity. In the field of water purification and environmental remediation, the polymerized MXene matrix composite has a larger specific surface area, more functional groups, and better light absorption [37]. Therefore, MXene-based materials can rapidly and efficiently adsorb heavy metal ions and radionuclides in wastewater, becoming a new type of excellent adsorbent in the field of wastewater treatment. Furthermore, such materials can degrade the organic pollutants in air and wastewater in the form of photocatalysts [45]. MXene can also be combined with other substances with high electron transfer efficiency to form composite materials and has become a hot material for domestic and international research [46,47,48].

Due to its excellent electrochemical properties, it can be used as an ideal carrier for biomolecules, such as enzymes, antibodies, and aptamers, and has also been applied in exosome detection. Zhang et al. prepared a sensing platform capable of recognizing multiple biomarkers and exosomes using Cy3 fluorescent groups labeled with aptamers, applying the principle that MXene naturally quenches fluorescence and that the addition of exosomes restores fluorescence [49]. Zhang et al. used MXene nanosheets as loading carriers for loading more aptamers for detecting exosomes. Moreover, MXene can significantly amplify the electrogenerated chemiluminescence signal to construct an electrochemiluminescent biosensor [50]. The aforementioned excellent conductive and catalytic properties of MXene materials make them promising for applications in electrochemical sensing. However, until now, the characteristics of MXene nanoflakes, namely, easy stacking and oxidation, also limit their wider application. Although the performance of MXene material has not reached the theoretically determined levels, its strong development potential is beyond doubt. The scope for future development includes developing new preparation methods, synthesizing MXene with better physical and chemical properties, and combining other materials to make composites.

**Scheme 1:**
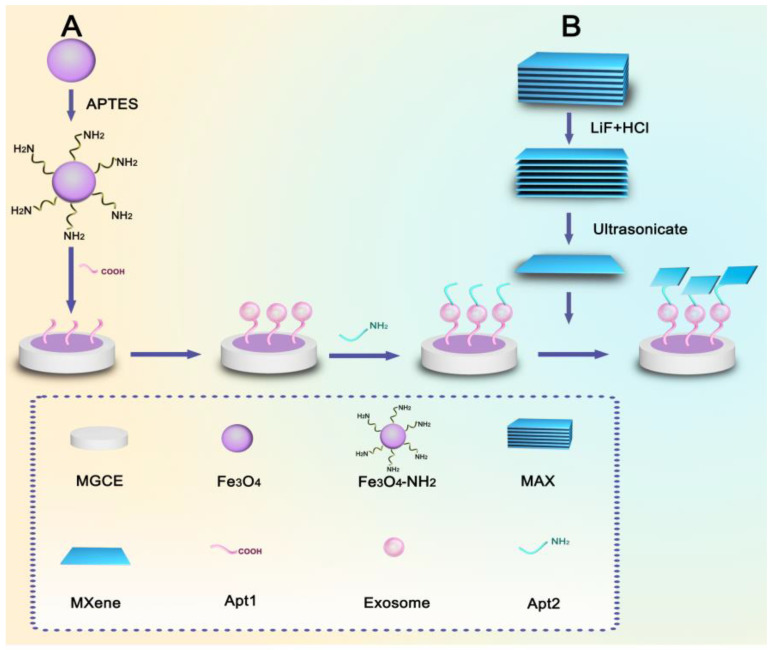
Schematic illustration of the electrochemical biosensor for exosome detection.

In this work, a novel sandwich-type electrochemical biosensor was developed for sensing exosomes based on amino-functionalized Fe_3_O_4_ nanoparticles (Fe_3_O_4_-NH_2_) modified magnetic glassy carbon electrode (MGCE) and two-dimensional Ti_3_C_2_ MXene nanosheets. As shown in Scheme 1, CD63 protein aptamer-labeled Fe_3_O_4_-NH_2_ nanoparticles were applied as the active interface for capturing exosomes. The Fe_3_O_4_-NH_2_ nanoparticles can recognize and capture target exosomes with the attachment of a CD63 aptamer. The as-synthesized Ti_3_C_2_ MXene nanosheets, with a large specific surface area and excellent electrical conductivity, were fabricated using the wet etching method as an electrochemical signal amplifier to enhance sensing performance. Due to the synergistic effect of MXene nanosheets anchored by the aptamer and magnetic adsorption of Fe_3_O_4_, a dual signal amplification strategy was realized, and excellent performance was exhibited with high sensitivity and reliability in exosome quantification. Consequently, the designed biosensor utilized two biomarkers to discriminate four different tumor cells (4T1, Hela, A549, and HepG2) and to improve the accuracy of validation, which can also be applied to the analysis of relevant serum samples. This aspect may provide a valuable tool for the expression of different exosomal proteins and the clinical diagnosis of tumors.

## 2. Materials and Methods

### 2.1. Reagents and Materials

Titanium aluminum carbide (Ti_3_AlC_2_) was purchased from 11 Technology Co., Ltd. (Jilin, China). LiF was obtained from Shanghai Aladdin Reagent Company (Shanghai, China). Iron (III) chloride hexahydrate (FeCl_3_·6H_2_O) and sodium acetate anhydrous (NaAc) were purchased from Shanghai Titan Scientific Co., Ltd. (Shanghai, China), and (3-(dimethyl-amino)propyl)-3-ethylcarbodii midehydrochloride (EDC), N-hydroxysuccinimide sodium salt (NHS), and (3-Aminopropyl)triethoxysilane (APTES) were obtained from Adamas Reagent Co., Ltd. (Shanghai, China). Poly (4-styrenesulfonic acid-co-maleic acid) sodium salt (PSSMA, n4-styrenesulfonic acid:nmaleic acid = 1:1) was purchased from Shanghai Macklin Biochemical Co., Ltd. (Shanghai, China). Hydrochloric acid (HCl) was obtained from Shanghai Lingfeng Chemical Reagent Co., Ltd. (Shanghai, China). The sequence of the capturing aptamer1 for CD63 on exosomes was 5′–COOH–TTTTTTCACCCCACCTCGCTCCCGTGACACTAATGCTA, the sequence of the probe aptamer2 for EpCAM on exosomes was 5′–NH_2_–TTTTTTCACTACAGAGGTTGCGTCTGTCCCACGTTGTCATGGGGGGTTGGC-CTG, and the sequence of the random aptamer3 was 5′–COOH–TTTTTTACACATTACAGGGGTTCGGTCTGGAAAGCAGTTACTGTCCCCTTGGGT. All of them were provided by Shanghai Sangon Biological Engineering Technology & Services Co., Ltd. (Shanghai, China). Ethylene glycol (EG) was purchased from Sinopharm Chemical Reagent Co., Ltd. (Shanghai, China). The magnetic glassy carbon electrodes (Φ = 3 mm) were obtained from Tianjin Incole Technology Co., Ltd. (Tianjin, China). The 100 K 15 mL ultrafiltration centrifugal tubes were purchased from Merck Milliporemerck Millipore Equipment Co., Ltd. (Shanghai, China).

### 2.2. Apparatus

The transmission electron microscopy (TEM) images were obtained using a transmission electron microscope H-7800 (Hitachi, Japan). The X-ray diffraction (XRD) patterns were collected using a D8 Advance (Bruker, Germany) X-ray diffractometer with Cu Kα radiation (λ = 1.5418 Å). The X-ray photoelectron spectroscopy (XPS) analyses were collected using an AXIS ULTRA DLD (Kratos, England). The scanning electron microscopy (SEM) images were collected using a Regulus 8100 Field emission scanning electron microscope (Hitachi, Japan). The saturation magnetization curve (VSM) was recorded with a vibrating sample magnetometer model 7404 (Lakeshore, Carson, CA, USA). The Fourier transform infrared (FT-IR) spectra were measured using a Nicolet is50 (Thermo Scientific, Waltham, MA, USA). Nanoparticle Tracking Analysis (NTA) was conducted using the NanoSightNS300 (Malvern Instruments, Worcestershire, England). Dynamic light scattering (DLS) and zeta potential measurements were performed using a Zetasizer Nano ZS-90 (Malvern, England).

The cyclic voltammetry (CV), differential pulse voltammetry (DPV), electrochemical impedance spectroscopy (EIS), and chronoamperometry (i-t) measurements were collected with a CHI660e (Shanghai Chenghua Instrument, Shanghai, China). The initial voltage and final voltage of the CV and DPV tests were set as −0.2 and 0.6 V, respectively. Moreover, the scan rate of CV tests was set as 0.1 V/s. EIS applied an AC voltage amplitude of 5 mV in a frequency range of 0.1 Hz to 100 kHz. Chronoamperometry (i-t) was conducted for 12,000 s with an initial voltage of 0.2 V. All electrochemical measurements used the conventional three-electrode system, which comprises three parts in a 5 mM K_3_[Fe(CN)_6_] solution as the redox probe solution with 0.1 M KCl. The modified magnetic glassy carbon electrodes were used as the working electrodes. A platinum wire and an Ag/AgCl served as the counter and reference electrodes, respectively.

### 2.3. Synthesis of Fe_3_O_4_-NH_2_

Synthesis of Fe_3_O_4_ nanospheres. The synthesis of Fe_3_O_4_ nanoparticles was based on the typical hydrothermal method. First, 0.55 g of PSSMA powder was dissolved into 20 mL of ethylene glycol (EG) under magnetic stirring until the solution reached transparency. Subsequently, 0.54 g of FeCl_3_·6H_2_O, 1.5 g of sodium acetate anhydrous (NaAc), and 8 mg of vitamin C were added in turn to form a homogeneous solution. The obtained reddish-brown solution was encapsulated in the autoclave and maintained at 200 °C for 10 h. After cooling to room temperature, the precipitate was washed with deionized water and ethanol alternately using magnetic separation six times to obtain pristine Fe_3_O_4_ nanoparticles.

Synthesis of Fe_3_O_4_-NH_2_. To obtain well-dispersed nanoparticles, the aforementioned Fe_3_O_4_ nanoparticles were dispersed in 6 mL of DI water with ultrasonication for 15 min. Magnetic spheres were redispersed in 100 mL of alcohol. Moreover, 0.4 mL of APTES was added into the solution under mechanical stirring and refluxing at 80 °C for 24 h. Finally, the product Fe_3_O_4_-NH_2_ was washed and then dried at 40 °C for future use.

### 2.4. Synthesis and Modification of MXene (Ti_3_C_2_)

Synthesis of MXene nanosheets. The synthesis of single-layer nanosheets Ti_3_C_2_ has been achieved using etching of the MAX phase in an HCl/LiF mixture. Briefly, 1.66 g of LiF was dissolved in 20 mL of HCl (9 M) with stirring at room temperature for 10 min. Subsequently, 1 g of MAX powders (Ti_3_AlC_2_) was added to the aforementioned mixture solution and reacted at 50 °C for 24 h. The product was repeatedly centrifuged with deionized water to pH = 6, and deionized water was then added to the Ti_3_C_2_ sediment for ultrasonication for 2 h. Finally, it was centrifuged in a centrifuge for 1 h to obtain a uniform Ti_3_C_2_ colloidal solution.

Synthesis of MXene-Gly hybrids. The procedure to prepare MXene-Gly was as follows. First, 10 mg of Gly was dispersed in 20 mL of deionized water with continuous stirring for 24 h. Next, 10 mL of Ti_3_C_2_ (1 mg/mL) was added to the Gly solution for 24 h under stirring. After two centrifugations, the precipitate obtained was dried in a vacuum freeze dryer.

### 2.5. Cell Culture, Exosome Extraction, and Counting

Mouse breast carcinoma cells (4T1 cells), human lung carcinoma cells (A549 cells), human cervical carcinoma cells (Hela cells), and human liver carcinoma cells (HepG2 cells) were cultured in Medium RPMI 1640 and DMEM with 10% FBS and 1% penicillin/streptomycin in a humidified atmosphere with 5% CO_2_ at 37 °C. Resuscitated cells grew to 90–95% and were then subcultured. Cells after the third generation can be used for exosome extraction. When the cells were cultured to the third generation and the cell fusion rate reached 70%, the original culture medium was removed and converted into a serum-free culture medium. When the cell fusion rate was 90–95%, the supernatant was taken to extract the exosomes.

Exosomes were purified and extracted using ultracentrifugation and ultrafiltration centrifugation, and the method was as follows. First, 20 mL of cell supernatant was centrifuged at 1000 rpm for 5 min at 4 °C to remove cells. Next, the cell supernatant was centrifuged at 2000× *g*, 4000× *g*, and 12,000× *g* for 30 min at 4 °C to remove cell debris and large vesicles. After being filtered with a 0.22 μm membrane, the aforementioned liquid was ultrafiltrated using a 100 KD ultrafiltration tube at 4000× *g* for 30 min at 4 °C. Finally, the liquid in the outer layer of the ultrafiltration tube was absorbed, diluted with PBS, and then stored at −20 °C for use.

### 2.6. Preparation of the Probe and Construction of the Electrochemical Sensor Platform

First, the MGCE was polished on suede with 0.05 μm aluminum oxide (Al_2_O_3_) powder and washed with deionized water until the potential difference was less than 80 mV (in 5 mM [Fe(CN)_6_]^3-/4-^ containing 0.1 M KCl), which could provide a stable electrical signal.

Synthesis of capture probe (Fe_3_O_4_-NH_2_-Apt1). The prepared Fe_3_O_4_-NH_2_ solution was taken and diluted to 1 mg/mL by adding deionized water. Moreover, the magnetic electrode was placed in 200 μL Apt1 (1 μM) for 2 h. Next, 100 μL of the prepared Fe_3_O_4_-NH_2_ solution was dropped into the aforementioned suspension for 30 min. Furthermore, 100 μL of EDC and NHS were added with incubation overnight at 4 °C. In this process, due to the role of magnetic adsorption, a large number of Fe_3_O_4_-NH_2_ spheres were adsorbed on the electrode surface for synthesizing amide bonds to obtain Fe_3_O_4_-NH_2_-Apt1/MGCE. Next, the modified electrodes were carefully rinsed with DI water. Exosomes derived from 4T1 cells were captured on Fe_3_O_4_-NH_2_-Apt1/MGCE by immersing the prepared electrodes in 1 mL of exosomes suspension at certain concentrations for 2 h at room temperature. Accordingly, exosomes/Fe_3_O_4_-NH_2_-Apt1/MGCE was successfully constructed.

Synthesis of detection probe (MXene-Gly-Apt2). The modified electrodes were immersed in 200 μL Apt2 (1 μM) for 2 h at room temperature to obtain Apt2/exosomes/Fe_3_O_4_-NH_2_-Apt1/MGCE. The modified electrodes were then immersed in 100 μL of the obtained MXene-Gly complex for 30 min at room temperature. Next, 100 μL of EDC and NHS were added into the solution and reacted at 4 °C overnight to obtain MXene-Gly-Apt2. For further adhesion, MXene-Gly was connected to achieve the complete sensor construction of MXene-Gly-Apt2/exosomes/Fe_3_O_4_-NH_2_-Apt1/MGCE. Finally, the aforementioned electrode was transferred into the electrolyte for electrochemical detection.

## 3. Results

### 3.1. Characterization of Fe_3_O_4_-NH_2_ and Ti_3_C_2_ MXene Nanosheets

**Figure 1:**
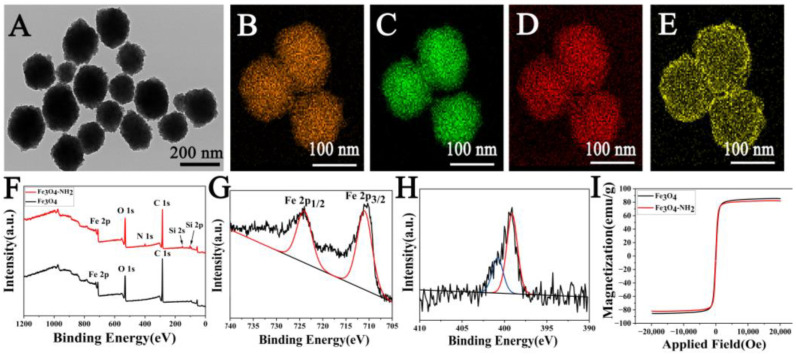
(**A**) TEM image of Fe_3_O_4_-NH_2_. (**B**–**E**) Elemental mapping of Fe, O, N, and Si in Fe_3_O_4_-NH_2_. (**F**) XPS patterns of Fe_3_O_4_ (a) and Fe_3_O_4_-NH_2_ (b). XPS spectra of (**G**) Fe 2p and (**H**) N 1s in the Fe_3_O_4_-NH_2_. (**I**) VSM of Fe_3_O_4_ before (a) and after (b) amination.

Fe_3_O_4_ nanoparticles were synthesized using the solvothermal method, in which EG and PSSMA were used as the solvent and dispersant, respectively, to make the synthesized products uniform in size and dispersed. The TEM image illustrated that Fe_3_O_4_ cores had a mean diameter of 200 nm (Figure S1A). After the modification of amination, the diameter of Fe_3_O_4_-NH_2_ increased slightly (Figure 1A). The dates of DLS and zeta potential also demonstrated that the target product Fe_3_O_4_-NH_2_ had been obtained because of the variation in electrical properties and dimensions. Considering Table S1, the particle size of Fe_3_O_4_ increased after amination. The bare magnetic spheres were negatively charged. However, amination changed their charge from negative to positive. Compared with the bare magnetic spheres, the N element of Fe_3_O_4_-NH_2_ appeared in the XPS patterns (Figure 1F). In Figure S2, the EDX spectrum with mapping of Fe_3_O_4_-NH_2_ further verified the existence of the N element. The saturation magnetization values of Fe_3_O_4_ and Fe_3_O_4_-NH_2_ were shown in Figure 1I. The value reduced slightly compared with that before amination.

**Figure 2:**
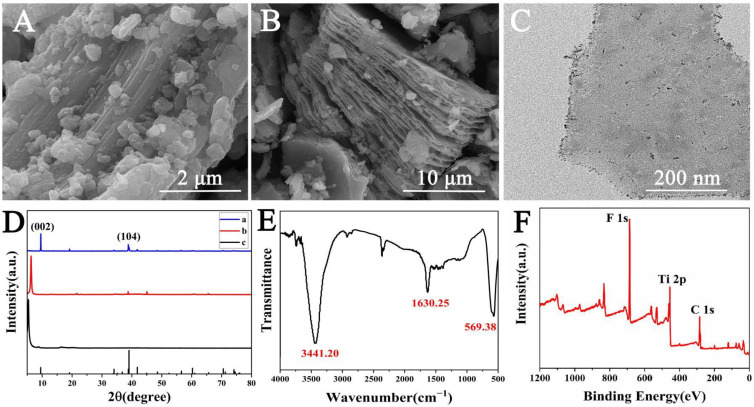
SEM images of (**A**) Ti_3_AlC_2_ and (**B**) Ti_3_C_2_. (**C**) TEM image of the MXene flake. (**D**) XRD patterns of MAX (a), delaminated MXene (b), and single-layer MXene (c). (**E**) FT-IR spectrum and (**F**) XPS pattern of the MXene nanosheets.

The synthesis of Ti_3_C_2_ MXene was realized using a wet etching process to remove the Al atomic layer from the MAX precursor with the etchant of the HCl/LiF mixture. The structural characteristics of the as-prepared MXene were clarified using SEM and TEM. Compared with Figure 2A, the accordion-like delaminated Ti_3_C_2_ in Figure 2B showed the successful etching of Al. Next, delaminated Ti_3_C_2_ was ultrasonically layered into single-layer or few-layer MXene nanosheets with a diameter of approximately 600 nm, which was characterized using TEM (Figure 2C). The DLS data verified that the Z-Average (d.nm) was 747.5 nm (Figure S3B). Furthermore, the XRD pattern was used to verify the qualitative analysis with regard to the successful synthesis of MXene. Compared with the peak position and peak intensity of the standard card (JCPDS No.52-0875), the raw material MAX phase Ti_3_AlC_2_ was consistent. Note that the (002) peak in the etched MXene shifted to the left, which also corresponded to the shift in peak position from 9.536° to 6.317°. According to the formula $\left(2\mathrm{dsin}\theta =n\lambda \right)$, the layer spacing of multi-layer MXene after etching was 2.09 nm. Furthermore, the (104) peak located at 38.915° gradually disappeared, illustrating the increase in layer spacing and the disappearance of the Al element, which confirmed the successful preparation of MXene (Figure 2D). Figure 2E showed the characteristic peaks in MXene, including O-H at ~3341 cm^−1^, C=O at ~1630 cm^−1^, and Ti-O at ~569 cm^−1^. Meanwhile, the results of XPS also verified the success of etching. In Figure 2F, the characteristic peaks in Ti 2p, C 1s, and F 1s were measured in the XPS patterns. The peak splitting diagram of MXene demonstrated the existence of Ti-O 2p_3/2_ (456.9 eV), Ti-O 2p_1/2_ (462.8 eV), Ti-C 2p_3/2_ (454.6 eV), and Ti-C 2p_1/2_ (458.3 eV). All results indicate the successful synthesis of MXene flakes (Figure S4A).

### 3.2. Electrochemical Behaviors of Ti_3_C_2_ MXene Nanosheets and Fe_3_O_4_-NH_2_

**Figure 3:**
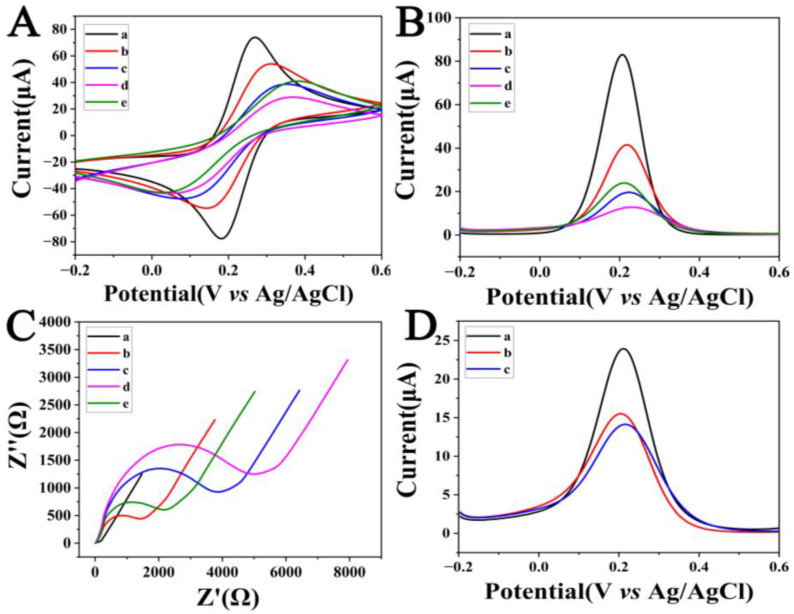
(**A**) Cyclic voltammograms, (**B**) differential pulse voltammetry, and (**C**) electrochemical impedance spectra of the electrode assembly process. Bare MGCE (a), Fe_3_O_4_-NH_2_-Apt1/MGCE (b), exosomes/Fe_3_O_4_-NH_2_-Apt1/MGCE (c), Apt2/exosomes/Fe_3_O_4_-NH_2_-Apt1/MGCE (d), and MXene-Gly-Apt2/exosomes/Fe_3_O_4_-NH_2_-Apt1/MGCE (e) in 5 mM [Fe(CN)_6_]^3-/4-^ containing 0.1 M KCl solution. The scan rate was 100 mV s^−1^. The impedance spectra were recorded from 0.1 Hz to 100 kHz with a signal amplitude of 5 mV at the formal potential of the redox couple. The concentration of exosomes was 10^4^ particles μL^−1^. (**D**) DPV current comparison of MXene-Gly-Apt2/exosomes/Fe_3_O_4_-NH_2_-Apt1/MGCE (a), MXene-Gly-Apt2/exosomes/Apt1/MGCE (b), and Gly-Apt2/exosomes/Fe_3_O_4_-NH_2_-Apt1/MGCE (c). The concentration of exosomes was 10^4^ particles μL^−1^.

The CV, DPV, and EIS tests mutually verified the whole process of electrode surface assembly. Fe(CN)_6_^3−/4−^ was used as an electrochemical probe to detect the change in CV curves during modification. As shown in Figure 3A, the bare electrode exhibited a pair of well-defined current peaks (curve a). After modifying the Fe_3_O_4_-NH_2_-Apt1 composite, the peak current dropped owing to the influence of Apt1 (curve b). Although Fe_3_O_4_-NH_2_ had excellent conductivity to amplify the electrochemical signal, the electrochemical signal was generally reduced because of the hindrance of Apt1 to electron transfer on the electrode surface. When the exosomes were incubated with the electrode, the electronic inertia of the exosomes further reduced the current peak (curve c). By binding Apt2 to exosomes and modifying it on the electrode interface, the negative charge of DNA repelled the negatively charged redox probe Fe(CN)_6_^3−/4−^ ions through the electrostatic repulsion effect (curve d). Finally, MXene was connected to the electrode surface, which improved the electron transfer rate and increased the conductivity of the electrode to improve the peak current significantly (curve e).

As shown in Figure 3B, the current curves of DPV achieved more sensitive and accurate measurements in which the change in current peak values correspond to those in corresponding CV curves. The layer-by-layer assembly of the electrode surface was further confirmed with the charge transfer resistance Rct of electrodes, as depicted in Figure 3C. The diameter of the semicircle in the Nyquist plot indicated the electron transfer resistance (Ret). Its change trend was consistent with those of the CV and DPV curves, which were mutually verified, thus proving the successful assembly of the biosensor.

To demonstrate the effect of the electrochemical performance on Fe_3_O_4_-NH_2_ and MXene-loaded electrodes, Fe_3_O_4_-NH_2_ and MXene were used to detect exosomes, respectively. As shown in Figure 3D, the electrochemical signal was significantly reduced if MXene and Fe_3_O_4_-NH_2_ were used alone compared with the simultaneous use of MXene and Fe_3_O_4_-NH_2_, indicating that the simultaneous loading of both materials can maximize the amplification of the electrochemical signal and achieve sensitive detection of exosomes.

### 3.3. Optimization of the Designed Biosensor

The optimal conditions for the biosensor were obtained by varying six aspects: the concentrations of Apt1, Apt2, MXene, and Fe_3_O_4_-NH_2_ and the incubation temperature and time. In the concentration range of 0.5 μM to 2 μM, the current peaked at 1 μM (Apt1), as illustrated in Figure S7A. Additionally, 1 μM was the ideal concentration for Apt2 (Figure S7B). Meanwhile, the concentration of the MXene solution was also adjusted to change the electrode. With the increase in MXene concentration, the sensor performance reached the optimal value when the concentration was 1 mg/mL (Figure S7C). During the incubation, large electrochemical signals were obtained at both 37 °C and room temperature, with the optimal temperature being room temperature (Figure S7D). In Figure S7E, the peak current maximized at 120 min from the beginning of the incubation period. When the concentration of Fe_3_O_4_-NH_2_ was 1mg/mL, the current peak was maximized (Figure S7F). Therefore, the operating conditions for this experiment were selected as follows: 1 μM of Apt1, 1μM of Apt2, 1 mg/mL of MXene solution, incubation time of 2 h at room temperature, and 1mg/mL of Fe_3_O_4_-NH_2_.

### 3.4. Exosome Sensitivity and Specificity of Biosensor

**Figure 4:**
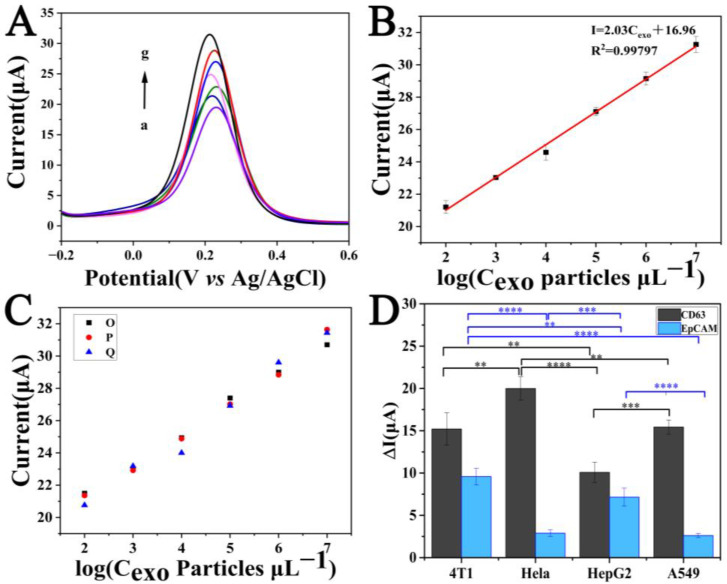
(**A**) DPV current of different exosome concentrations (a–g: 0, 10^2^, 10^3^, 10^4^, 10^5^, 10^6^, and 10^7^ particles μL^−1^). (**B**) Plot of DPV intensity vs. the logarithm value of the derived exosome concentration of 4T1 cells. (**C**) DPV current of three repeated experiments at different exosome concentrations from 10^2^ to 10^7^ particles μL^−1^. (**D**) DPV current of two types of cancer markers (CD63 and EpCAM) from the solution with different exosome lines (4T1, Hela, HepG2, and A549) at a concentration of 10^5^ particles μL^−1^. **: *p* < 0.01; ***: *p* < 0.001; ****: *p* < 0.0001. Black symbol “*” represents CD63 and blue symbol “*” represents EpCAM.

To realize the quantitative detection of the electrochemical sensor, the DPV signal was recorded by changing the concentration of 4T1 exosomes under optimal conditions. The results of the current curves were shown in Figure 4A, and the measurement range was expanded from 10^2^ particles μL^−1^ to 10^7^ particles μL^−1^. The linear equation is $I=2.03{\log[C}_{\mathrm{exo}}{(\mathrm{exo}\;\mu L}^{-1})]+16.96{(R}^{2}=0.99797)$ with the detection limit of 43 particles μL^−1^ (S/N = 3), where I is the current peak measured using the electrochemical sensor of 4T1 exosomes at a specific concentration and log C_exo_ is the logarithm of the concentration of the exosomes (Figure 4B).

Additionally, samples containing a random sequence aptamer (Apt3) and devoid of exosomes were utilized as controls to show the specificity of the produced electrochemical biosensor. The results were shown in Figure S8. The peak current of the random sequence sample was clearly lower than that of the sample incubated with exosomes at a concentration of 10^4^ particles μL^−1^ and was only marginally higher than that of the sample without exosomes. It indicated that in this case, almost no exosomes can bind to the electrode surface, resulting in MXene being unable to amplify the current signal.

### 3.5. Exosome Repeatability, Reproducibility, and Stability of the Biosensor

Repeatability is also rated as one of the most critical requirements for sensor performance measurements. In Figure 4C, three replicate experiments were performed for each concentration. The relative standard deviations (RSDs) obtained ranged from 0.57% to 2.13%. The minimum RSD was 0.57% when the concentration of exosomes was 10^3^ particles μL^−1^. The reproducibility of the constructed biosensor was shown in Figure S9. In nine parallel experiments using exosomes extracted from different batches, the DPV current maintained a relatively stable value with an RSD of 2.87%. The stability of the biosensor was also studied. From 400 s to 12,000 s, the DPV current values were so stable that the current variation range was controlled within 7.5% (Figure S10). The aforementioned results demonstrated the favorable stability of the biosensor. The findings revealed a steady overall trend and a few minor deviations, illustrating the feasibility of the sensor.

### 3.6. Detection Analysis of Different Exosomes and Practical Samples

Exosomes have various surface proteins, such as CD63, CD81, CD9, and EpCAM. However, the amount of surface proteins in exosomes secreted by different cells varies. Therefore, the content of surface proteins can be used to determine the cell type of the exosomes, which can be applied clinically for early screening of cancer. The proposed electrochemical biosensor used two surface proteins (CD63 and EpCAM) as detection markers to distinguish four different types of cancer cells (4T1, Hela, HepG2, and A549). Compared with a single marker, it can improve detection stability and accuracy.

In Figure 4D, compared with HepG2 cells, the contents of CD63 in exosomes of 4T1 and A549 were significantly higher, while the highest content of CD63 protein was found in exosomes of Hela. Since the difference in the CD63 content on the exosomal surfaces of 4T1 and A549 cells was insignificant, the content of the EpCAM protein was used for further distinction. Obviously, the exosomes of 4T1 cells contain more EpCAM than A549 cells. Meanwhile, Hela and HepG2 exosomes both had variable amounts of the EpCAM protein. This result also confirmed the universal expression of CD63 protein on the surface of exosomes [51]. The CD63 protein content on the cell surface was defined as the difference between the current peak after Apt1 binding to the electrode surface and the incubation of exosomes. The EpCAM protein content on the surface of exosomes was determined as the difference between the current peak after the binding signal amplifier and incubation of exosomes. The findings show that the developed electrochemical biosensor can assess the amounts of protein expression for the aforementioned four exosomes in cells, providing a foundation for clinical study, clinical cancer diagnosis, and follow-up cancer therapy.

Further, to initially verify the potential of the sensor for application in real samples, four different concentrations of exosomes were added to the serum for electrochemical detection. The obtained results were listed in Table 1, with recoveries ranging from 101% to 110% at four different concentrations from 10^3^ particles μL^−1^ to 5 × 10^4^ particles μL^−1^. The aforementioned results may be due to the influence of the exosomes in serum on the sensor. However, this still proves that the designed sensor is sensitive to exosomes and can be applied to corresponding clinical detection.

## 4. Conclusions and Discussion

In summary, a novel electrochemical biosensor was proposed with Fe_3_O_4_-NH_2_-Apt1 as the capture probe to amplify the electrochemical signal while increasing the binding amount of the electrode surface and improving the capture efficiency. Meanwhile, the MXene nanosheets were two-dimensional materials with excellent conductivity for further amplifying the electrochemical signal. The whole strategy easily and effectively generated and amplified electrical signals, detected 4T1 exosomes in the linear range of 10^2^ particles μL^−1^–10^7^ particles μL^−1^ with a detection limit of 43 particles μL^−1^, and realized the identification of four tumor cells with dual protein markers. The detection capability of the sensor was also validated in serum samples.

Further, the next study can focus on real samples from patients with multiple tumors and whether the period of cancer can be determined by analyzing and comparing the differences in exosomal surface protein expression in the early and middle stages of tumors. Moreover, different synthetic methods for MXene produce different materials with different defects and terminal groups, which affect the electrochemical performance of the prepared MXene. Density functional theory predicts that surface termination strongly affects the Fermi level density of MXene states, thus affecting the electronic conductivity of MXenes [52]. Some reports have shown that the electrical conductivity of MXene-containing surface terminal groups (-OH and -F) decreases [53]. In addition, different layer spacings and specific surface areas also lead to the difference in ion diffusion ability. Among them, intercalation can increase the MXene resistivity of five atomic layers by an order of magnitude [54]. Therefore, the structure and properties of synthetic MXene must be further explored to obtain the materials with the best performance to improve the detection sensitivity. The current study provides some reference and significance for the study of exosomal surface proteins and early non-invasive screening of cancer in clinical settings.

## Figures and Tables

**Table 1:** Exosome analysis in serum using the biosensor.

Sample Number	Added (log Particles μL^−1^)	Found (log Particles μL^−1^)	Recovery (%)
1	3.00	3.32	110
2	3.50	3.68	105
3	4.00	4.25	106
4	4.50	4.56	101

## Data Availability

Not applicable.

## Supplementary Materials

The following supporting information can be downloaded at: https://www.mdpi.com/article/10.3390/s23073508/s1, Figure S1: The TEM and SEM characterizations of Fe_3_O_4_ nanospheres; Figure S2: The EDX characterizations of Fe_3_O_4_ nanospheres; Figure S3: The DLS characterization of MXene nanosheets; Figure S4: The XPS characterizations of MXene nanosheets; Figure S5: The FT-IR and XPS characterizations of MXene-Gly; Figure S6: The characterizations of exosomes; Figure S7: The optimal conditions for the biosensor; Figure S8: The specificity of the constructed biosensor; Figure S9: The reproducibility of the constructed biosensor; Figure S10: The stability of the constructed biosensor; Table S1: Comparison of Fe_3_O_4_ and Fe_3_O_4_-NH_2_ on DLS and zeta potential; Table S2: Comparison of the performance of the designed sensors [55,56].
