# Supplementary material for: High-Performance Detection of Exosomes Based on Synergistic Amplification of Amino-Functionalized Fe3O4 Nanoparticles and Two-Dimensional MXene Nanosheets
Source: Sensors (Basel). 2023 Mar 27;23(7):3508. doi: 10.3390/s23073508 (PMC10099274; doi:10.3390/s23073508)
Supplement: Supplementary file 1 [file sensors-23-03508-s001.zip › sensors-2247816-supplementary.pdf]

Supplementary Material

High-performance detection of exosomes based on synergistic amplification of amino-functionalized Fe_3O_4 nanoparticles and two-dimensional MXene nanosheets

Linlin Zhuang [1,2], Qiannan You [1,2*], Xue Su [1,2], Zhimin Chang [2], Mingfeng Ge [2], Qian Mei [2], Li Yang [3*], Wen-Fei Dong [1,2], Li Li [1,2*]

[1] School of Biomedical Engineering (Suzhou), Division of Life Sciences and Medicine, University of Science and Technology of China, Hefei 230026, P. R. China

[2] Suzhou Institute of Biomedical Engineering and Technology, Chinese Academy of Science, Suzhou 215163, P. R. China

[3] Chongqing Institute of Green and Intelligent Technology, Chinese Academy of Sciences, Chongqing 400714, P. R. China

Corresponding Author

[*] E-mail:

youqn@sibet.ac.cn;

ylyhp@126.com;

lil@sibet.ac.cn.

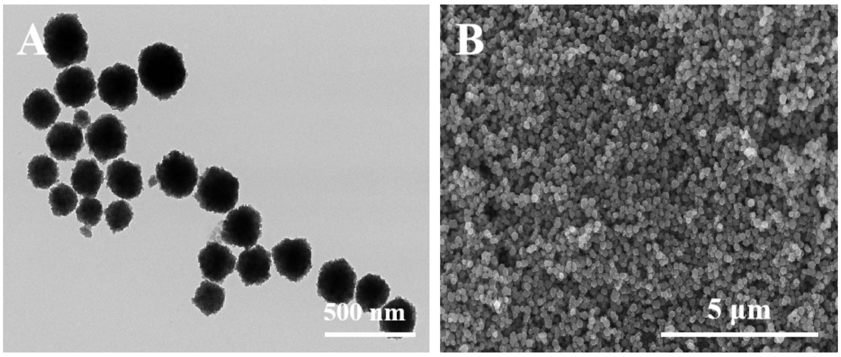

Figure S1 (A) TEM image of Fe_3O_4 nanospheres. (B) SEM image of Fe_3O_4 nanospheres.

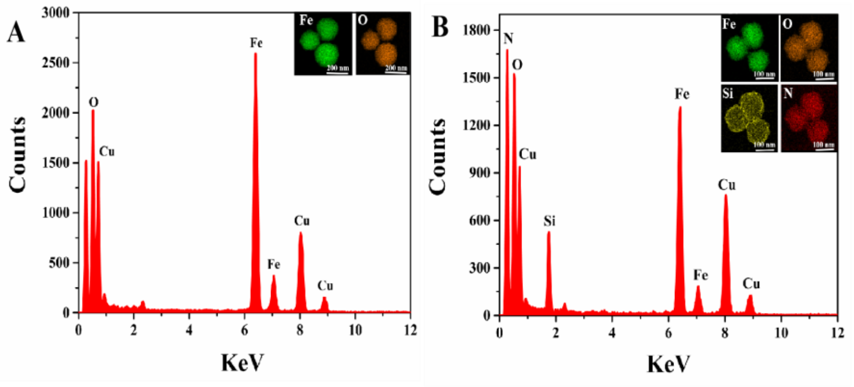

Figure S2 (A) EDX spectrum with mapping of Fe_3O_4 nanospheres. (B) EDX spectrum with mapping of Fe_3O_4-NH_2 nanospheres.

Table S1.

Comparison of Fe_3O_4 and Fe_3O_4-NH_2 on DLS and zeta potential.

Sample	Zeta（mV）	Size（nm）
Fe_3O_4	-39.3	214.5
Fe_3O_4-NH_2	+27.9	222.8

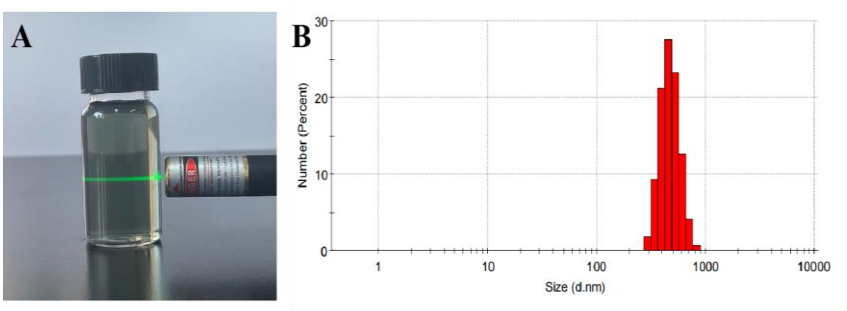

Figure S3 Photograph of the MXene colloid solution (A) and particle size distribution of MXene (B).

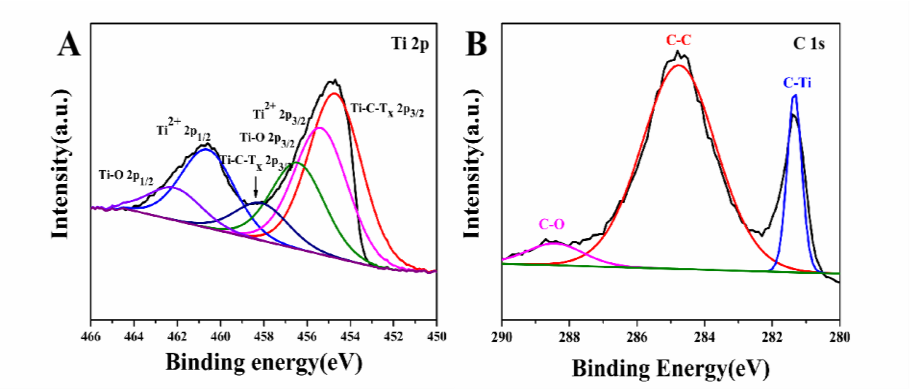

Figure S4 (A) XPS spectrum of MXene in Ti 2p. (B) XPS spectrum of MXene in C 1s.

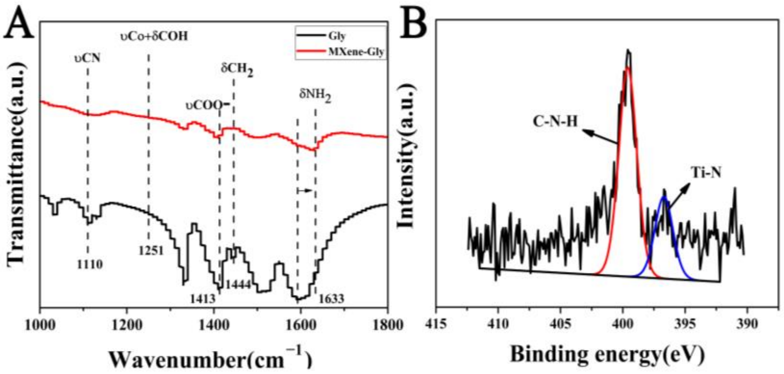

Figure S5 (A) FT-IR analysis of MXene-Gly and Gly. (B) XPS spectrum of MXene-Gly in N 1s. The linkage of amino groups on MXene was realized by MXene-Gly.

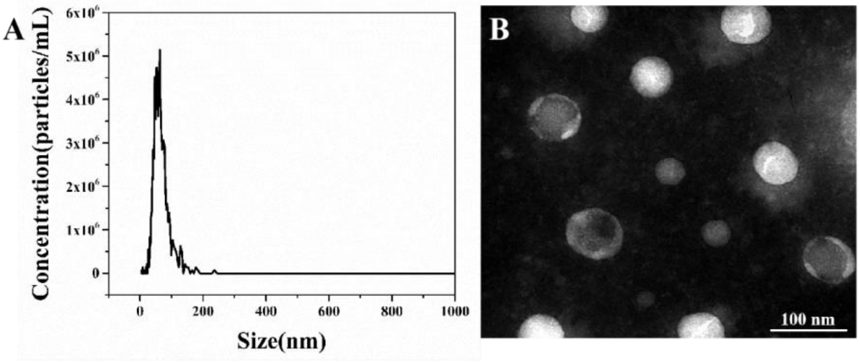

Figure S6 (A) The NTA data of exosomes derived from 4T1 cells. (B) TEM image of exosomes isolated from the culture medium of 4T1 cells.

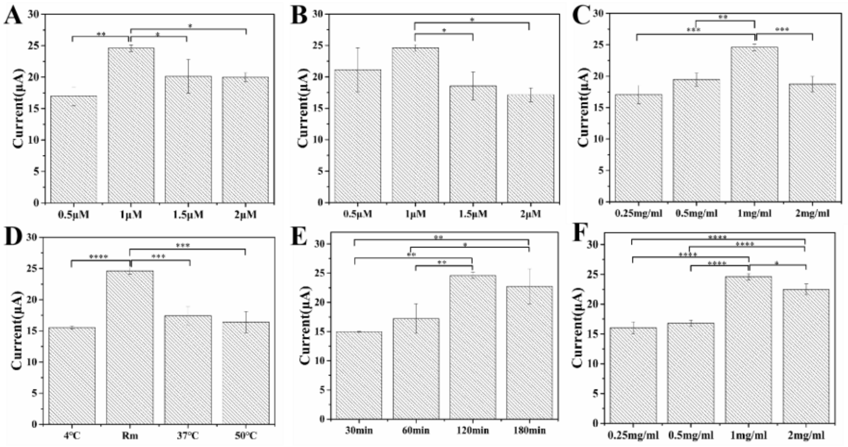

Figure S7 (A) The DPV current of the biosensor tested with different concentrations of Apt1, (B) different concentrations of Apt2, (C) different concentrations of MXene, (D) different incubation temperatures of exosomes, (E) different incubation time of exosomes and (F) different concentrations of Fe_3O_4-NH_2. The concentrations of exosomes were 10^4 particles μL^{-1}. The error bars were calculated from three times parallel experiments (*: $p< 0.05$; **: $p< 0.01$; ***: $p < 0.001$; ****: $p < 0.0001$).

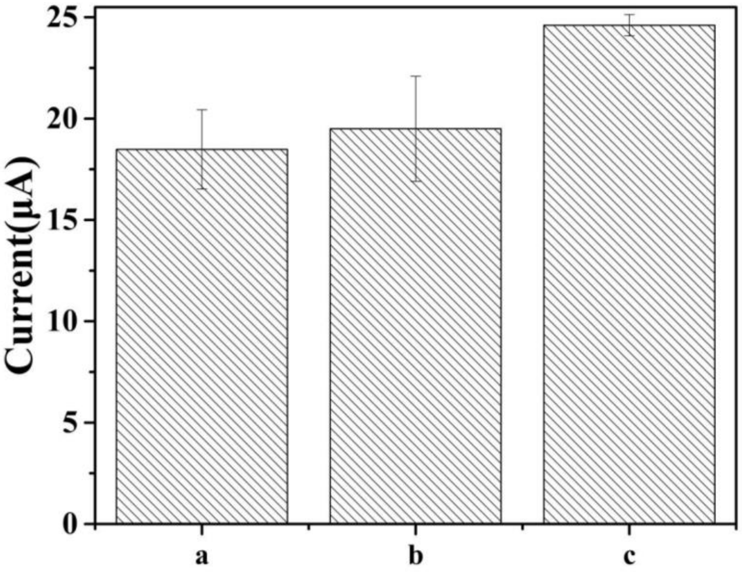

Figure S8 The DPV current of MXene-Gly-Apt2/Fe_3O_4-NH_2-Apt1/MGCE (a), MXene-Gly-Apt2/exosomes/Fe_3O_4-NH_2-Apt3/MGCE (b), MXene-Gly-Apt2/exosomes/ Fe_3O_4-NH_2-Apt1/MGCE (c). The concentration of exosomes was 10^4 particles μL^{-1}.

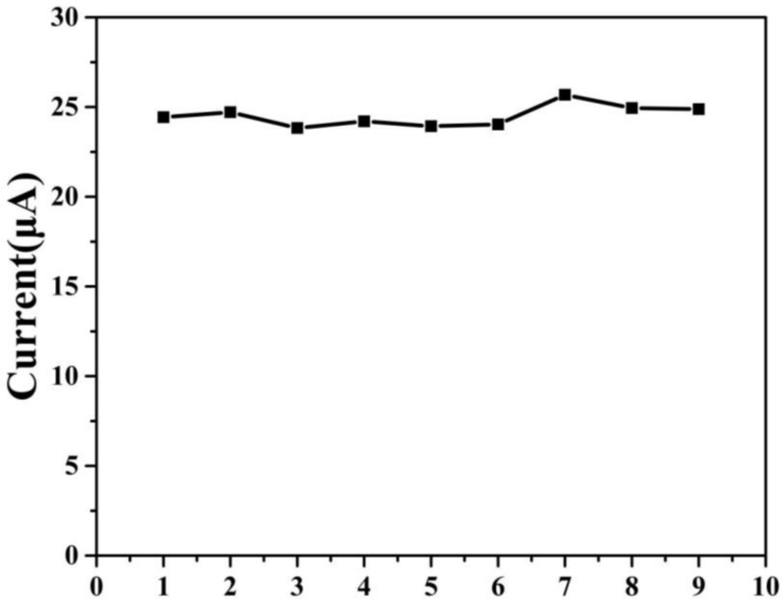

Figure S9 The DPV current of 9 parallel tests with exosomes extracted in different batches. The concentration of exosomes was 10^4 particles μL^{-1}.

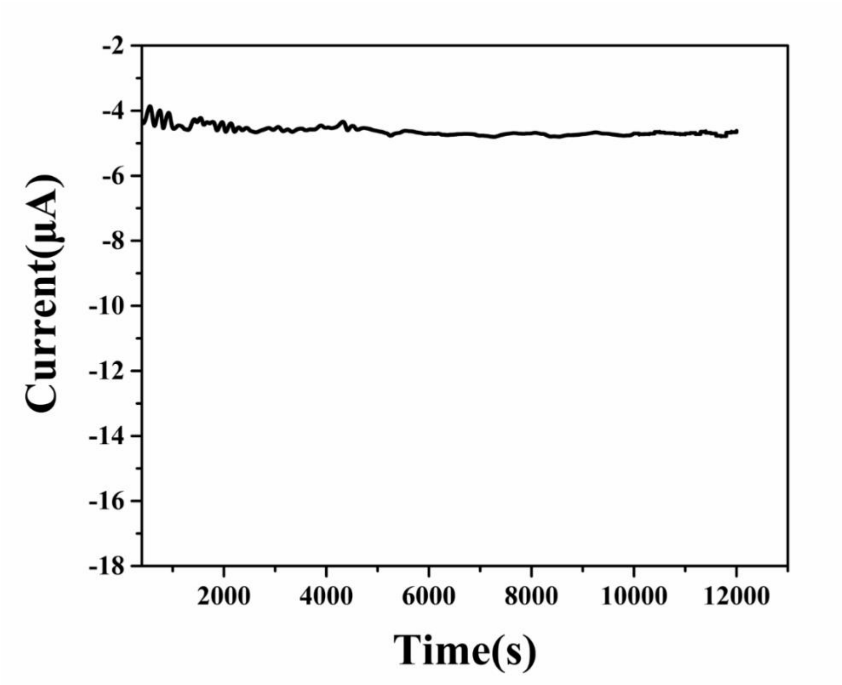

Figure S10 The i-t curve of exosomes obtained from 4T1 cells at room temperature in 0.2V for 12000s. The concentration of exosomes was 10^4 particles μL^{-1}.

Table S2.

The performance of the designed detection platform for detecting exosomes is compared with other reported work.

Materials	Strategy	Detection limit （particles/μL）	Linear range (particles/μL)	Ref
Ti_3C_2	Electrochemical	229	5×10^2-5×10^5	[22]
Ti_3C_2	Fluorescence	1.4×10^3	10^4-10^9	[50]
MXenes-BPQDs	Chemiluminescence	37	1.1×10^2-1.1×10^7	[55]
AuNPs-Ti_3C_2	Chemiluminescence	30	10^2-10^5	[56]
Fe_3O_4-Ti_3C_2	Electrochemical	43	10^2-10^7	This work

References

22. Zhang, H.; Wang, Z.; Wang, F.; Zhang, Y.; Wang, H.; Liu, Y. Ti3C2 MXene mediated Prussian blue in situ hybridization and electrochemical signal amplification for the detection of exosomes. *Talanta* **2021**, *224*, 121879, doi:10.1016/j.talanta.2020.121879.

50. Zhang, Q.; Wang, F.; Zhang, H.; Zhang, Y.; Liu, M.; Liu, Y. Universal Ti3C2 MXenes Based Self-Standard Ratiometric Fluorescence Resonance Energy Transfer Platform for Highly Sensitive Detection of Exosomes. *ANALYTICAL CHEMISTRY* **2018**, *90*, 12737-12744, doi:10.1021/acs.analchem.8b03083.

55. Fang, D.; Zhao, D.; Zhang, S.; Huang, Y.; Dai, H.; Lin, Y. Black phosphorus quantum dots functionalized MXenes as the enhanced dual-mode probe for exosomes sensing. *Sensors and Actuators B: Chemical* **2020**, *305*, 127544, doi:10.1016/j.snb.2019.127544.

56. Zhang, H.; Wang, Z.; Wang, F.; Zhang, Y.; Wang, H.; Liu, Y. In Situ Formation of Gold Nanoparticles Decorated Ti(3)C(2) MXenes Nanoprobe for Highly Sensitive Electrogenerated Chemiluminescence Detection of Exosomes and Their Surface Proteins. *Anal Chem* **2020**, *92*, 5546-5553, doi:10.1021/acs.analchem.0c00469.

Commented [M1]: Citations in the Supplementary Materials should also be included in the main references (otherwise they will not count towards citations metrics—including the impact factor calculations—for the cited work). If references in individual supplementary files are put in the main text, all of the references must have a citation in the “Supplementary Materials” section (e.g., “References [x,x] are cited in the supplementary materials”) or in main text.

Commented [2R1]: After checking, we have added references in individual supplementary files to the main text with modifying their number.

22.

50.

55.

56.
